# Nanostructured Oxide (SnO_2_, FTO) Thin Films for Energy Harvesting: A Significant Increase in Thermoelectric Power at Low Temperature

**DOI:** 10.3390/mi15020188

**Published:** 2024-01-26

**Authors:** Karuppiah Deva Arun Kumar, S. Valanarasu, Alex Capelle, Sibel Nar, Wael Karim, Arnaud Stolz, Barthélemy Aspe, Nadjib Semmar

**Affiliations:** 1 Groupe de Recherches sur l’Énergétique des Milieux Ionisés, GREMI, Université d’Orléans, CNRS, 14 Rue d’Issoudun, 45067 Orléans, Francesibel.nar@univ-orleans.fr (S.N.); wael.karim@univ-orleans.fr (W.K.); barthelemy.aspe@univ-orleans.fr (B.A.); 2 Department of Physics, Arul Anandar College, Madurai 625514, India; 3 Laboratoire Nanotechnologies et Nanosystèmes (LN2)-CNRS IRL-3463, Université de Sherbrooke, Sherbrooke, QC J1K OA5, Canada

**Keywords:** oxide thin films, spray pyrolysis, Hall Effect, Seebeck coefficient

## Abstract

Previous studies have shown that undoped and doped SnO_2_ thin films have better optical and electrical properties. This study aims to investigate the thermoelectric properties of two distinct semiconducting oxide thin films, namely SnO_2_ and F-doped SnO_2_ (FTO), by the nebulizer spray pyrolysis technique. An X-ray diffraction study reveals that the synthesized films exhibit a tetragonal structure with the (200) preferred orientation. The film structural quality increases from SnO_2_ to FTO due to the substitution of F^−^ ions into the host lattice. The film thickness increases from 530 nm for SnO_2_ to 650 nm for FTO films. Room-temperature electrical resistivity decreases from (8.96 ± 0.02) × 10^−2^ Ω·cm to (4.64 ± 0.01) × 10^−3^ Ω·cm for the SnO_2_ and FTO thin films, respectively. This is due to the increase in the carrier density of the films, (2.92 ± 0.02) × 10^19^ cm^−3^ (SnO_2_) and (1.63 ± 0.03) × 10^20^ cm^−3^ (FTO), caused by anionic substitution. It is confirmed that varying the temperature (K) enhances the electron transport properties. The obtained Seebeck coefficient (*S*) increases as the temperature is increased, up to 360 K. The synthesized films exhibit the *S* value of −234 ± 3 μV/K (SnO_2_) and −204 ± 3 μV/K (FTO) at 360 K. The estimated power factor (PF) drastically increases from ~70 (μW/m·K^2^) to ~900 (μW/m·K^2^) for the SnO_2_ and FTO film, respectively.

## 1. Introduction

Energy resource depletion has become one of the most crucial problems in the modern world, which demands the use of energy in different forms. Factories, automobiles, steel industries, and other similar sources produce about ~75% of waste heat [1], which is very difficult to recycle. Hence, it is our responsibility to explore alternatives for reusing waste heat energies from diverse sources. Thermal energy harvesting offers a promising approach to harness the abundance of freely available heat and transform it into a more practical and usable form, such as electrical energy. The performance of a thermoelectric (TE) material is expressed by the dimensional figure of merit *zT* = (*σS*^2^/*κ*) × *T*, where *σ* is the electrical conductivity, *S* is the Seebeck coefficient, *κ* is the thermal conductivity, and *T* is the absolute temperature [2]. Some commercial chalcogenide-based thermoelectric alloy materials such as Pb, Hg (toxic), Bi, Te, Ag, and Se (expensive) are available for energy harvesting. However, alloys are unstable in air and can decompose at quite low temperatures. Additionally, the electrical conductivity, Seebeck coefficient, and thermal conductivity cannot be tuned independently.

Oxide materials are known to be highly promising for practical operations such as optoelectronics, photovoltaics, thermoelectrics, etc. In addition, oxide materials are more stable at high temperatures and are inexpensive. Researchers have therefore focused on the fabrication of stable, eco-friendly, abundant, and cost-effective TE devices based on oxide materials. Bulk oxide materials have some disadvantages, however, such as the time required for the sintering process, fabrication of the *n*-type and *p*-type elements, arrangement of device shapes, and mechanical fragility. In contrast, oxide thin films offer substantial advantages compared to bulk oxide materials such as quick fabrication, flexibility, and control of defects even at the nanoscale.

Many oxide thin films such as cadmium oxide (CdO), zinc oxide (ZnO), titanium dioxide (TiO_2_), tin dioxide (SnO_2_), etc., are available for various device fabrications. However, SnO_2_ thin films can outperform the electronic transport behavior of other oxide films due to their intrinsic defects and good environmental stability. SnO_2_ is a familiar *n*-type semiconducting metal oxide, and it has a wide bandgap (~3.6 eV) with low binding energy (~30 meV) [3]. There are some impurities or doping elements that can be incorporated or substituted in the SnO_2_ system without affecting the host lattice structure; this could improve the electrical properties of SnO_2_. Fluorine (F^−^) is the main doping element considered for SnO_2_ to form an FTO system, as the electrical performance is expected to be significantly increased [4]. In the FTO system, an anionic source of fluorine ions (F^−^) replaces O^2−^; thus, fluorine could act as a free electron. Reports show that FTO has a higher electron carrier density, wide band gap, and high transparency in the visible region [5]. Alternatively, Sn-doped indium oxide (In_2_O_3_:Sn, ITO) has also attracted the attention of the research community because of its wide range of practical applications. Nevertheless, in comparison to FTO, ITO is a more expensive material. Additionally, the substitution of Sn^2+^ ions can affect the host In^3+^ ions, leading to a reduction in carrier concentration and electron mobility.

Based on the literature, Bian Tian et al. [6] reported that the average Seebeck coefficient (*S*) value of ITO/In_2_O_3_ is about 109 μV/°C at high temperature. This value is relatively lower than the one reported by Ferreira et al. [7] for the SnO_2_ thin film, who found that the Seebeck coefficient was −255 μV/K with a low power factor (~10^−4^ W/m·K^2^) at room temperature. On the other hand, G. Gordillo et al. [8] studied F-doped SnO_2_ thin films by spray pyrolysis at 400 °C. While incorporating HF into the host precursor (Sn) solution, they noticed a reduced electrical resistivity at 3 × 10^−4^ Ω·cm. However, under the same conditions, the Seebeck coefficient (thermoelectric power) also exhibited a low value, measuring less than −100 μV/K. Therefore, our goal is to enhance the Seebeck coefficient and power factor of F-incorporated SnO_2_ thin films for thermoelectric energy conversion, focusing on commercial aspects. 

Recent reports of the preparation of SnO_2_ and FTO thin films used different physical and chemical methods [7,8,9,10,11,12,13,14,15]. All of these methods have advantages and disadvantages. Among them, chemically grown thin film techniques are easy to handle and make large-area deposition possible. Nebulizer-assisted spray pyrolysis (NSP) stands out with a significant benefit: it offers a cost-effective, non-vacuum technique for large-area deposition, capable of producing high-quality films with minimal precursor volume. The NSP method operates based on the Bernoulli principle [16]. When a pressurized airflow is directed through a constricted orifice, the velocity of the airflow increases, generating a stream of mist particles. The stream of mist particles, known as aerosols (particle size ~2.5 μm), helps improve the quality of the film and enables uniform growth due to gradual nucleation with minimum wastage. The benefits of NSP methods are high productivity, ease of handling, controllable film growth rate, ability to achieve desired thickness, rapid fabrication over larger areas, deposition feasibility at both low and high temperatures, and the formation of pinhole-free films [3,17]. Based on the above-mentioned advantages, we used the NSP technique to develop good-quality SnO_2_-based thin films at the desired substrate temperature. 

The objective of this work is to study the effect of F^−^ ions on the thermoelectric properties of SnO_2_ thin films by the economic nebulizer-assisted spray pyrolysis technique. In this report, we investigate both SnO_2_ and FTO thin films to study their structural, morphological, and thermoelectric properties. Lastly, the Seebeck coefficient and power factor values obtained are compared to those reported in previous studies.

## 2. Materials and Methods

### 2.1. Thin Film Synthesis by Nebulizer-Assisted Spray Pyrolysis

High-purity precursors of anhydrous tin (II) chloride (SnCl_2_) and ammonium fluoride (NH_4_F) were purchased from Sigma Aldrich and Nice Chemicals, with 3 N purity. The optimum concentrations of tin (II) chloride (0.09 M) and ammonium fluoride (0.01 M or 10%) precursors were dissolved one by one in 10 mL of isopropanol and deionized water as a solvent in the ratio of 3:1 (isopropanol/DI water). The prepared solution was continuously stirred at room temperature for 15 min to form a homogeneously mixed solution.

**Figure 1:**
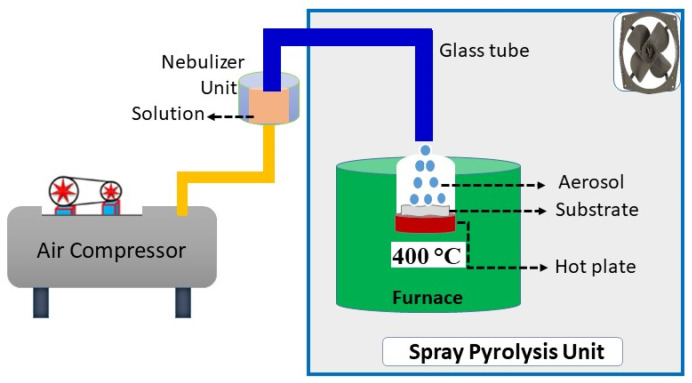
Schematic view of the nebulizer spray pyrolysis setup process.

Before the film preparation, borosilicate glass substrates (SiO_2_-B_2_O_3_) were cleaned using deionized water, isopropanol, and acetone, followed by drying in a hot-air oven at 200 °C for 2 h. The process is helpful to remove contamination or dust particles from the raw substrate, and to make impurity-free cleaned substrates. The pre-cleaned glass substrate was kept on the hot plate, which was maintained at 400 °C (±5 °C) [3]. The equipped solution was placed in the nebulizer container, which is well connected to the glass tube (spray gun). Figure 1 shows a schematic view of the nebulizer spray pyrolysis process at the desired temperature. The precursor solution was continuously sprayed (10 min) on the heated substrate in an X–Y direction to obtain uniform film formation, as well as good adherence over the substrate surface. In this study, we employed other optimized deposition parameters, maintaining a constant spray pressure of 1.5 kg/cm^2^ and a nozzle-to-substrate distance of 3 cm. After completing 10 mL of sprayed solution, the deposited films were allowed to cool at room temperature for further characterization.

### 2.2. Characterizations

The following tools were used to analyze the film’s structural, morphological, and thermoelectric properties. The structural properties of the films were determined by XRD using Cu-Kα radiation (Bruker D8 Discover, Billerica, MA, USA), and the morphological analysis was carried out on a scanning electron microscopy (SEM) system (Zeiss Supra 40, Oberkochen, Germany). The film thickness was measured using a stylus profilometer (Bruker Dektak, Frankfurt, Germany), and cross-sectional SEM imaging was also performed. Room-temperature Hall Effects were measured through four-probe measurements (ECOPIA-HMS5500, Republic of Korea). Temperature-dependent Seebeck coefficient (*S*) variation was systematically observed by using the Peltier module for temperature change monitoring, connected with a Keithley DMM6500, USA (homemade setup as in a previous study [18]). The measurement of the Seebeck coefficient was conducted in the temperature range from 300 K to 360 K. All the characterizations were performed at the GREMI laboratory, Orleans.

## 3. Results and Discussion

### 3.1. Structural Properties

**Figure 2:**
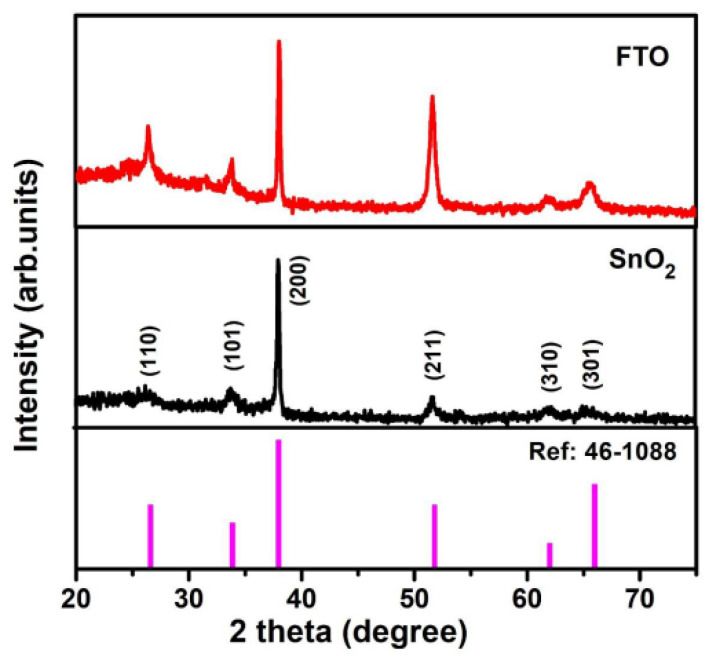
XRD patterns of the synthesized oxide (SnO_2_ and FTO) thin films; data compared with JCPDS card No: 46-1088 [19].

The XRD patterns of the synthesized oxide thin films are shown in Figure 2. From the XRD pattern, the six identified peaks were determined as corresponding to the (110), (101) (200), (211), (310), and (301) planes of the polycrystalline SnO_2_ structure, as the pattern matches with a reference file from the data base: JCPDS card No: 46-1088 [19]. It can be seen that the tetragonal phase structure is attained for both synthesized thin films. The SnO_2_ lattice incorporates doped F^−^ ions in interstitial positions, while simultaneously replacing O^2−^ ions with F^−^ ions for the FTO system [20]. No secondary phases related to Sn, F clusters, or SnO were detected in the XRD data, confirming that the films consist solely of the SnO_2_ phase. This result is likely attributed to the low doping concentration of F^−^ ions. In addition, no peak shift was observed in the XRD pattern despite doping. However, the full width at half maximum of the peaks decreased slightly from SnO_2_ to FTO thin films; this can be attributed to an improvement in the structural quality. In addition, with the inclusion of fluorine ions in the SnO_2_ lattice, an increase in the (211) preferred orientation compared with the strong (200) preferential orientation of the SnO_2_ film was observed. The relative peak intensity change in diffraction peaks might be due to the changes in the atomic distribution upon doping [21,22].

### 3.2. SEM Analyses

**Figure 3:**
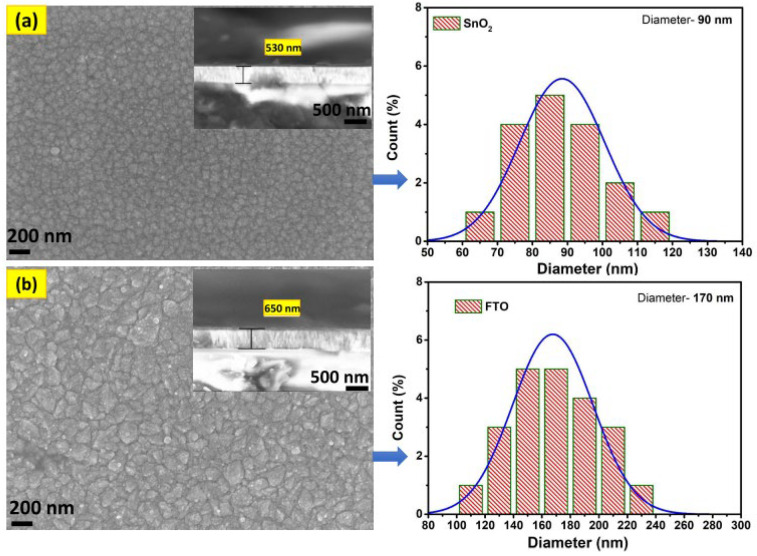
SEM images of the synthesized oxide thin films: (**a**) SnO_2_ and (**b**) FTO. The corresponding size distribution graphs are presented on the right side of each image. The SEM images include cross-sectional views to show the films’ thickness.

Morphological (SEM) analysis was used to investigate the grain growth level as well as the size of the grains for the synthesized films. Figure 3a,b displays the SEM images acquired for the oxide films, specifically for SnO_2_ and FTO, respectively. In Figure 3a, the SnO_2_ film displays small spherical-shaped grains distributed over the surface without any holes or cracks. With the inclusion of F^−^ ions in the host material, the spherical grains observed in the FTO thin film, as depicted in Figure 3b, exhibit an enlargement in size and interconnection with each other. The increase in grain size confirms the increase in grain growth when compared to pure SnO_2_. The particle distribution curves for both synthesized thin films, namely SnO_2_ and FTO, are depicted on the right side of Figure 3a,b, respectively. The estimated average grain size is ~90 nm for SnO_2_ and ~170 nm for FTO film, respectively. The increase in grain size can be attributed to the variation in film thickness. The film thickness increases from ~530 nm in SnO_2_ film to ~650 nm in FTO film, as validated by cross-sectional SEM images provided in the insert in Figure 3a,b. The increase in film thickness might be due to the effect of F-doping, allowing a higher concentration of dopants to be incorporated into the SnO_2_ lattice. This phenomenon appears to enhance both the carrier concentration (*n*_e_) and carrier mobility (*μ*_e_), and is discussed in the Hall measurement section. In general, any semiconducting materials should have a high carrier concentration (~10^20^ cm^−3^) because there is less scattering of electrons in the grains. In our case, the presence of spherically interconnected grains facilitates low electron scattering, primarily attributed to the ionized impurity scattering process [23,24].

In Figure S1 (Supplementary Materials), the EDX images illustrate the synthesized SnO_2_ and FTO films, along with the corresponding elemental mapping results. The EDX results affirm the existence of fluorine within the SnO_2_ lattice, showing that the oxygen ions are occupied by fluorine. The inserted table verifies the substitution of F^−^ ions into O^2−^ sites, which is clearly apparent as the elemental oxygen content decreases from the SnO_2_ to the FTO thin film. Figure S1 illustrates the mapping pictures correlating SnO_2_ and FTO films. Observable elements such as tin, oxygen, and fluorine are detected on the surface of the FTO thin film. Moreover, tin and oxygen, as host elements, exhibit a predominant arrangement on the SnO_2_ compared to the FTO film. Additionally, platinum is detected on the surface of both films; it was sputtered (4 nm) before measurement to prevent charging.

### 3.3. Hall Effect Measurements

Hall Effect measurement was performed to obtain electrical resistivity (*ρ*), electron carrier concentration (*n*_e_), and electron mobility (*μ*_e_) values for the synthesized oxide films, which are detailed in Table 1. Typically, SnO_2_ thin films exhibit a higher carrier concentration and low resistivity due to their intrinsic defects. Moreover, the introduction of F^−^ ions into the SnO_2_ lattice may modify its electrical performance. This is due to the reduction in grain boundaries causing the scattering of free electrons. In the FTO system, either tin or fluorine ions can randomly replace an intrinsic oxygen vacancy, resulting in the release of well-localized electrons [25] in the conduction band (CB) or in the lattice oxygen sites (O^2−^), thereby providing free electrons. This free electron generation in CB leads to an increase in the carrier concentration and reduces the resistivity. 

The enhanced electrical performance of the FTO system may be attributed to the following possible reasons: (i) oxygen (O^2−^) vacancies being occupied by fluorine (F^−^) ions, which generate free electrons within the system; (ii) substitutional incorporation of F^−^ ions into oxygen sites as extensively discussed in our prior report [3]. The electrical resistivity obtained is (8.96 ± 0.02) × 10^−2^ Ω·cm for SnO_2_ and decreases to (4.64 ± 0.01) × 10^−3^ Ω·cm for FTO films. This can be attributed to the ongoing occupation of oxygen vacancies by F^−^ ions, consequently increasing the carrier concentration from (2.92 ± 0.02) × 10^19^ cm^−3^ to (1.63 ± 0.03) × 10^20^ cm^−3^ for the respective films. The presence of accumulated grains (evidenced in SEM) could promote continuous moving paths for electrons and reduce the e^−^/h^+^ recombination [26]; hence, there is an increase in electron mobility. Here, the observed electron mobility rises from (2.41 ± 0.08) cm^2^/V·s to (8.40 ± 0.02) cm^2^/V·s for SnO_2_ and FTO films, respectively. Based on Hall Effect measurements, the FTO thin film exhibits a higher carrier concentration and lower resistivity than SnO_2_. 

### 3.4. Thermoelectric Properties

**Figure 4:**
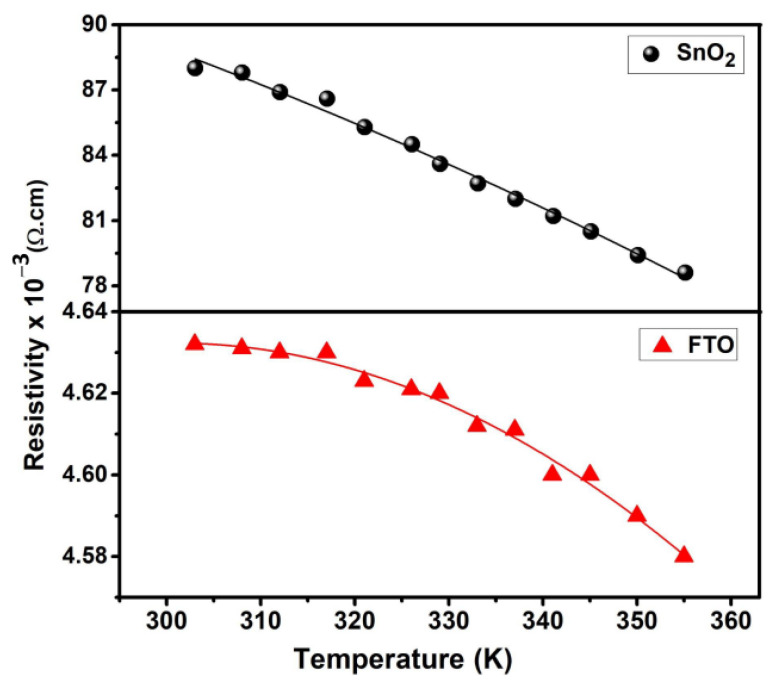
The temperature dependence of electrical resistivity (*ρ*) for the SnO_2_ synthesized thin films is represented by the upper curve, while the lower curve corresponds to FTO.

The present work aims to study the thermoelectric (TE) properties of cost-effective semiconducting oxide thin films fabricated with a nebulizer-assisted spray technique. The Peltier module-based homemade Seebeck coefficient measurement is presented in Figure S2 (Supplementary Materials). Two key parameters that play an important role in determining the TE performance are the resistivity (*ρ*) and the Seebeck coefficient (*S*). By using both *ρ* and *S* values, the power factor of the TE materials can be estimated by *S*^2^/*ρ* [27]. Figure 4 shows the variation in electrical resistivity as a function of temperature (300–360 K) for the synthesized films. The *ρ* value of both of the synthesized films decreases as a function of increasing temperature, attributed to the rise in electron mobility (*μ*_e_). Furthermore, this resistivity trend with operating temperature is ascribed to the decrease in activation energy. It can be seen that the minimum *ρ* is (4.58 ± 0.01) × 10^−3^ Ω·cm at 360 K compared to (4.64 ± 0.01) × 10^−3^ Ω·cm at 300 K for the FTO thin film. Table 1 lists the temperature-dependent *ρ*, *μ*_e_, and *n*_e_ values. It is evident that the FTO film resistivity (~10^−3^ Ω·cm) is relatively one order lower than that of the SnO_2_ film (~10^−2^ Ω·cm) due to the high electron concentration and mobility, as discussed in Section 3.4. Khelifi et al. [12] reported a similar electrical conductivity value of 8.11 × 10^2^ Ω·cm^−1^ for F-doped SnO_2_ thin films prepared by ultrasonic spray pyrolysis. 

The Peltier module was employed as a steady-state method to measure the Seebeck coefficient value of the synthesized thin films. With the help of the Peltier module for an accurate temperature change measurement, the Seebeck coefficients were determined using the following classical relations:

(1) $$\;\;\;S=\frac{dV}{dT}\;;\;\;\;\;\;dV=S\cdot dT$$

(2) $$\;\;\;\;\;\;\Delta V=\int_{T_{1}}^{T_{2}}S\;\left(T\right)\;dT\;\;;\;\;S\left(T=\frac{T_{1}+T_{2}}{2}\right)\;$$

(3) $$\;\;\int_{T_{1}}^{T_{2}}\left(S\right)dT=\int_{V_{1}}^{V_{2}}dV$$

(4) $$\;\;\;\;\;\;\;\Delta V=S\cdot \Delta T;\;\;S=\frac{\Delta V}{\Delta T}$$

where Δ*V* and Δ*T* represent the difference in electrical voltage and the temperature change, respectively [28]. *T*_1_ and *T*_2_ are the temperatures of the hot and cold ends, respectively. The heating sequence used to measure the synthesized FTO thin film is depicted in Figure S3 (Supplementary Materials), establishing Δ*T* with corresponding Δ*V*. 

**Figure 5:**
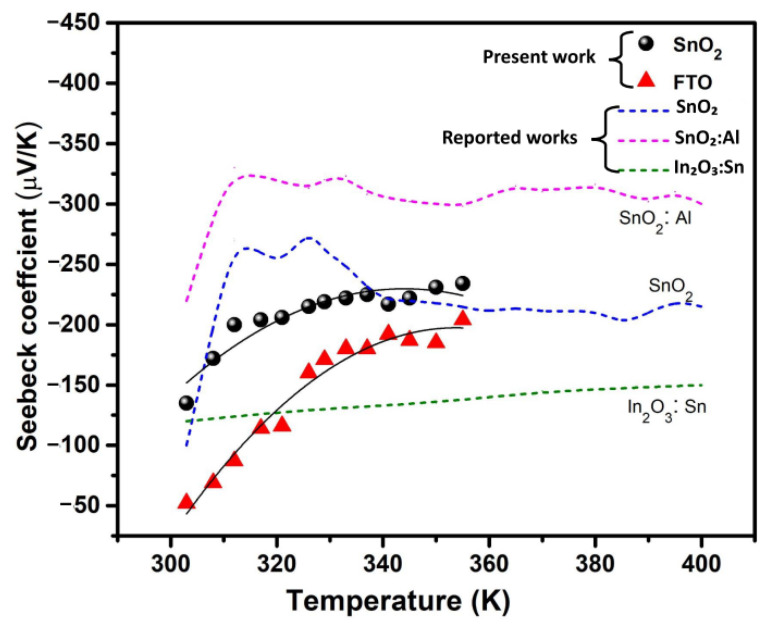
Temperature dependence of the Seebeck coefficient (*S*) for the synthesized thin films, and the variation in *S* compared with reported works [35,36,37].

Figure 5 shows the variation in the Seebeck coefficient as a function of temperature (300–360 K) for the synthesized films. In accordance with the literature, at room temperature, the SnO_2_ thin films show *n*-type conduction originating from ionized defects such as oxygen vacancies [29]. In our case, we noted negative *S* values in both synthesized thin films, confirming their classification as *n*-type semiconducting thermoelectric materials. The Hall measurement study provided additional support for this observation, revealing a negative Hall coefficient. Consequently, both the Seebeck and Hall measurements corroborate that both oxide films exhibit *n*-type behavior, indicating that electrons are the predominant charge carriers. In Figure 5, the Seebeck coefficient displayed a consistently negative sign across the entire temperature range, with increasing values of *S* indicating a decrease in the electron density. The *S* variation in both thin films increased with increasing temperature. The synthesized SnO_2_ film exhibited the *S* value of −135 ± 3 μV/K at 300 K and rose to a maximum of −234 ± 3 μV/K at 360 K. For the FTO film, a low *S* value was measured of −52 ± 3 μV/K at 300 K and −204 ± 3 μV/K at 360 K. According to the literature, *S* is inversely proportional to the *σ* as a function of carrier concentration *n*_e_ [30]. The absolute value of *S* is lower in the FTO film compared to the SnO_2_ film in the entire temperature range, possibly due to the difference in the grain boundary and oxygen deficiency. In addition, the lower *S* is consistent with the higher *σ* in Table 1 due to maximum *n*_e_. Although the SnO_2_ film has a larger *S* in the entire temperature range, this can be explained with the minimum *n*_e_. Yakuphanoglu [11] observed an *S* value of −500 μV/K at 300 K for a SnO_2_ thin film coated on an ITO substrate by dip coating, and Trejo-Zamudio et al. [31] reported that the maximum *S* value found was −200 μV/K at 300 K for a SnO_2_ thin film Bi-doped by spray pyrolysis, which is consistent with our measurements. In addition to this, several previously documented studies on SnO_2_ and dissimilar metal-doped SnO_2_ thin films [32,33,34] consistently align with our work in terms of their corresponding Seebeck coefficients. In Figure 5, we present a comparison of data from the reported studies on oxide thin films. The synthesized SnO_2_ film demonstrates an asymptotic behavior, closely matching the variation reported for the SnO_2_ film [35]. However, the reported film exhibits a maximum *S* in the temperature range from 310 K to 340 K, which could be attributed to the difference in film thickness (~450 nm), lower than our synthesized SnO_2_ film (~530 nm).

**Figure 6:**
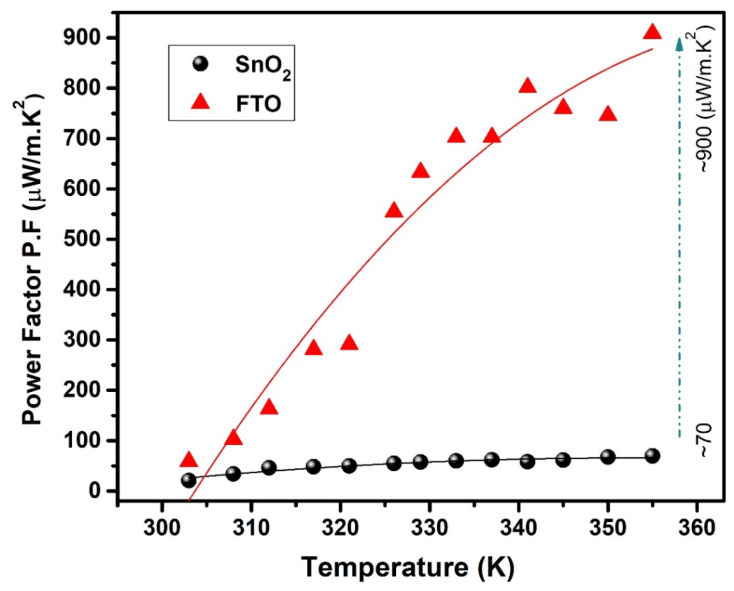
Temperature dependence of the power factor (PF) for the synthesized oxide thin films of SnO_2_ and FTO.

The estimated power factor (PF) is shown in Figure 6 as a function of temperature. The temperature-dependent PF was estimated by using both *ρ* and *S* values across the entire temperature range. It is evident that the Seebeck coefficient exhibits an increasing trend with rising temperature. As anticipated from the low *ρ* and high *S* values, the estimated PF consistently increases as the temperature rises up to 360 K. In the extrinsic conductivity region, the increase in PF with temperature is due to the increasing number of thermally excited charge carriers. The values of *ρ*, *S*, and PF obtained for the synthesized oxide films are listed in Table 1 and compared with the values reported in previous studies on oxide thin films [35,36,37], as presented in Table 2. At 360 K, the maximum power factor value is found for the FTO thin film is ~900 μW/m·K^2^, which is one order of magnitude higher than that of the SnO_2_ film (~70 μW/m·K^2^). The maximum achieved PF (~900 μW/m·K^2^) is noticeably higher when compared with the reported values for SnO_2_ thin films (1 × 10^−4^ W/m·K^2^) [7]. Ribeiro et al. [38] reported a higher PF value (~0.5 mW/mK^2^) for transparent oxide (TiO_2_:Nb) thin films (~150 nm thick) near room temperature (300 K). Therefore, we conclude that the obtained PF value (~900 μW/m·K^2^) is two times higher than the previously reported values mentioned above for different oxide thin films. The increase in PF is attributed to the substantial impact of the high Seebeck coefficient and electrical conductivity values, similar to the findings of our study. The powerful increase in PF up to 360 K suggests that the spray-pyrolysis-synthesized oxide thin films might be promising TE materials.

## 4. Conclusions

In this study, we examined the thermoelectric properties of nanostructured semiconducting oxide thin films, which were synthesized using a chemical spray pyrolysis technique at a substrate temperature of 400 °C. The oxide films exhibit a polycrystalline structure with a preferential orientation along the (200) plane of the tetragonal crystal structure as confirmed by XRD analysis. Upon F-doping, the resistivity of the SnO_2_ film decreased from (8.96 ± 0.02) × 10^−2^ Ω·cm to (4.64 ± 0.01) × 10^−3^ Ω·cm. This change in electrical transport behavior can be attributed to the introduction of fluorine dopants into the SnO_2_ lattice. The inclusion of F^−^ ions as dopants modulates the *ρ* and *S* of the synthesized film, influenced by both the structural crystallinity and film thickness. The low *ρ* and moderate *S* values obtained led to the achievement of a high PF by varying the temperature. The maximum PF of ~900 μW/m·K^2^ was achieved for the FTO thin film, which is one order of magnitude higher than that of SnO_2_ film (~70 μW/m·K^2^) at 360 K. The obtained PF value (~900 μW/m·K^2^) is two times higher than the previously reported values for different oxide thin films. With their remarkable properties, the chemically grown *n*-type oxide thin films become highly attractive materials for constructing future TE devices, particularly when combined with *p*-type materials for thermoelectric junction generators.

## Figures and Tables

**Table 1:** Temperature-dependent Hall Effect and TE parameters for the synthesized oxide thin films.

Electrical and TE Parameters	SnO_2_		FTO	
	300 K	360 K	300 K	360 K
*n* (cm^−3^)	(2.92 ± 0.02) × 10^19^	(2.52 ± 0.02) × 10^19^	(1.63 ± 0.03) × 10^20^	(1.56 ± 0.02) × 10^20^
*μ* (cm^2^/V·s)	2.41 ± 0.08	2.77 ± 0.04	8.40 ± 0.02	8.71 ± 0.02
*ρ* (Ω·cm)	(8.96 ± 0.02) × 10^−2^	(7.95 ± 0.03) × 10^−2^	(4.64 ± 0.01) × 10^−3^	(4.58 ± 0.01) × 10^−3^
*S* (μV/K)	−135 ± 3	−234 ± 3	−52 ± 3	−204 ± 3
PF (μW/m·K^2^)	~20	~70	~58	~900

**Table 2:** Comparative thermoelectric results between the synthesized oxide thin films and those reported previously.

Materials	*ρ *(mΩ·m)	*S* (µV/K)	PF (mW/m·K^2^)	Working Temp. (K)	Method	Reference
(i). SnO_2_ (ii). FTO	0.889 0.046	135–234 52–204	0.02–0.07 0.058–0.90	300–360 300–360	Nebulizer Spray Pyrolysis	Our results
SnO_2_	0.27	100–200	0.05–0.15	310–400	Spray Pyrolysis	[35]
SnO_2_	0.61	255	0.1	300	RF Sputtering	[7]
SnO_2_:Al (10%)	0.32	250	-	300	Spray Pyrolysis	[36]
In_2_O_3_:Sn (5%)	0.04	120–200	1.5–4.7	300–723	Spray Pyrolysis	[37]

## Data Availability

The data are included in the main text and in the Supplementary Information.

## Supplementary Materials

The following supporting information can be downloaded at: https://www.mdpi.com/article/10.3390/mi15020188/s1. Figure S1: EDX imaging records the synthesized thin films of SnO_2_ and FTO, along with elemental mapping images for each individual element; Figure S2: Peltier module-based homemade Seebeck coefficient measurement with the temperature range from 300 K to 360 K; Figure S3: Example of heating sequence for establishing Δ*T* in a temperature range from 22 °C to 28 °C, along with corresponding Δ*V* for FTO thin film.
