# Supplementary material for: Nanostructured Oxide (SnO2, FTO) Thin Films for Energy Harvesting: A Significant Increase in Thermoelectric Power at Low Temperature
Source: Micromachines (Basel). 2024 Jan 26;15(2):188. doi: 10.3390/mi15020188 (PMC10890522; doi:10.3390/mi15020188)
Supplement: Supplementary file 1 [file micromachines-15-00188-s001.zip › micromachines-2769141-supplementary.pdf]

Nanostructured Oxide (SnO_2, FTO) Thin Films for Energy Harvesting: A Significant Increase in Thermoelectric Power at Low Temperature

Karuppiah Deva Arun Kumar [1,2], S. Valanarasu[2], Alex Capelle[1], Sibel Nar[1,3], Wael Karim[1], Arnaud Stolz[1], Barthélemy Aspe[1], Nadjib Semmar[1]**

[1]Groupe de Recherches sur l'Énergétique des Milieux Ionisés, GREMI, Université d'Orléans, CNRS, 14 Rue d'Issoudun, 45067, France
[2]Department of Physics, Arul Anandar College, - 625514, Madurai, India
[3]Laboratoire Nanotechnologies et Nanosystèmes (LN2)-CNRS IRL-3463, Université de Sherbrooke, 3000 Boulevard Université, Sherbrooke, Québec, J1K OA5, Canada

Corresponding email: deva-arun-kumar.karuppiah@univ-orleans.fr & nadjib.semmar@univ-orleans.fr

Supplementary materials:

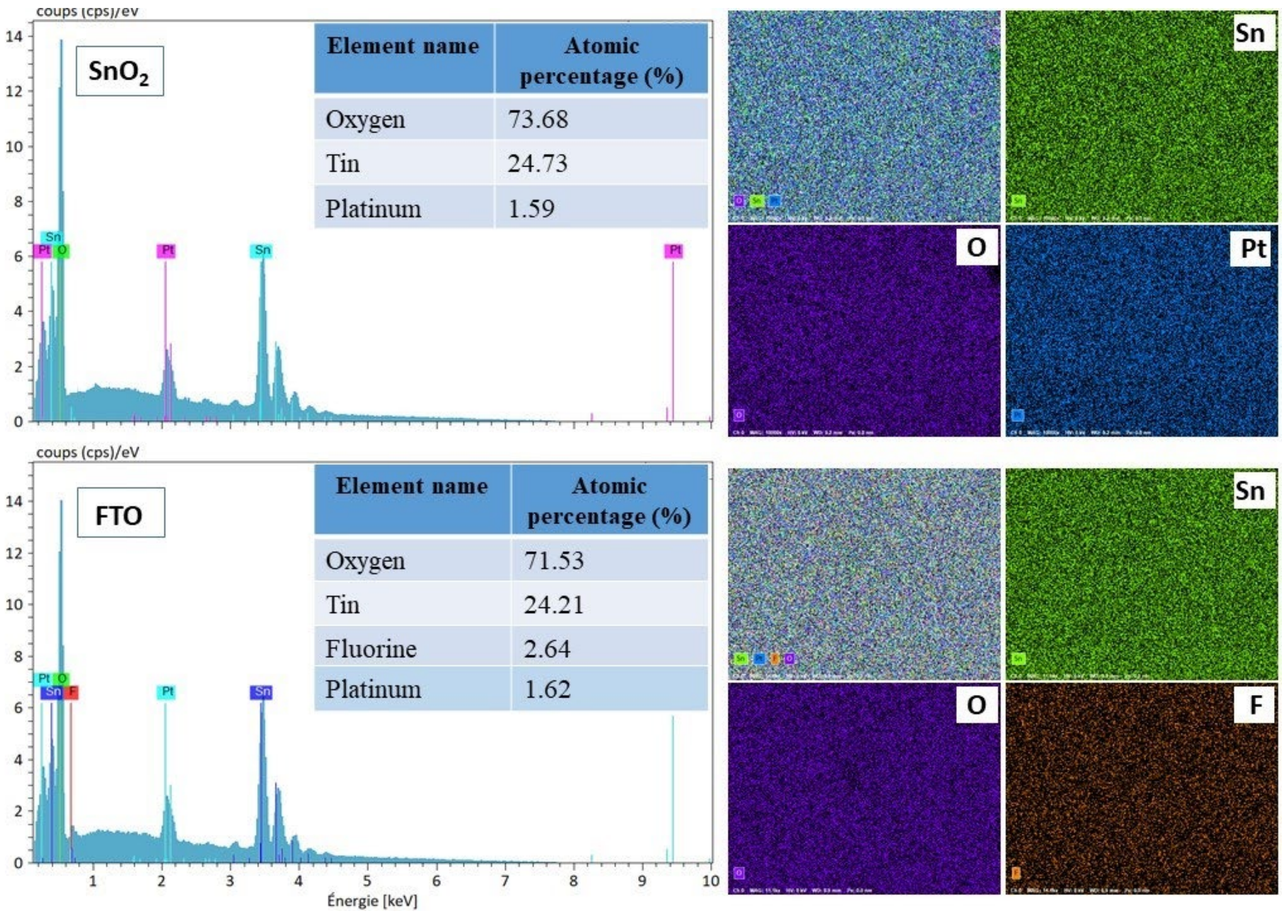

Figure S1 EDX imaging records the synthesized thin films of SnO_2 and FTO, along with elemental mapping images for each individual element.

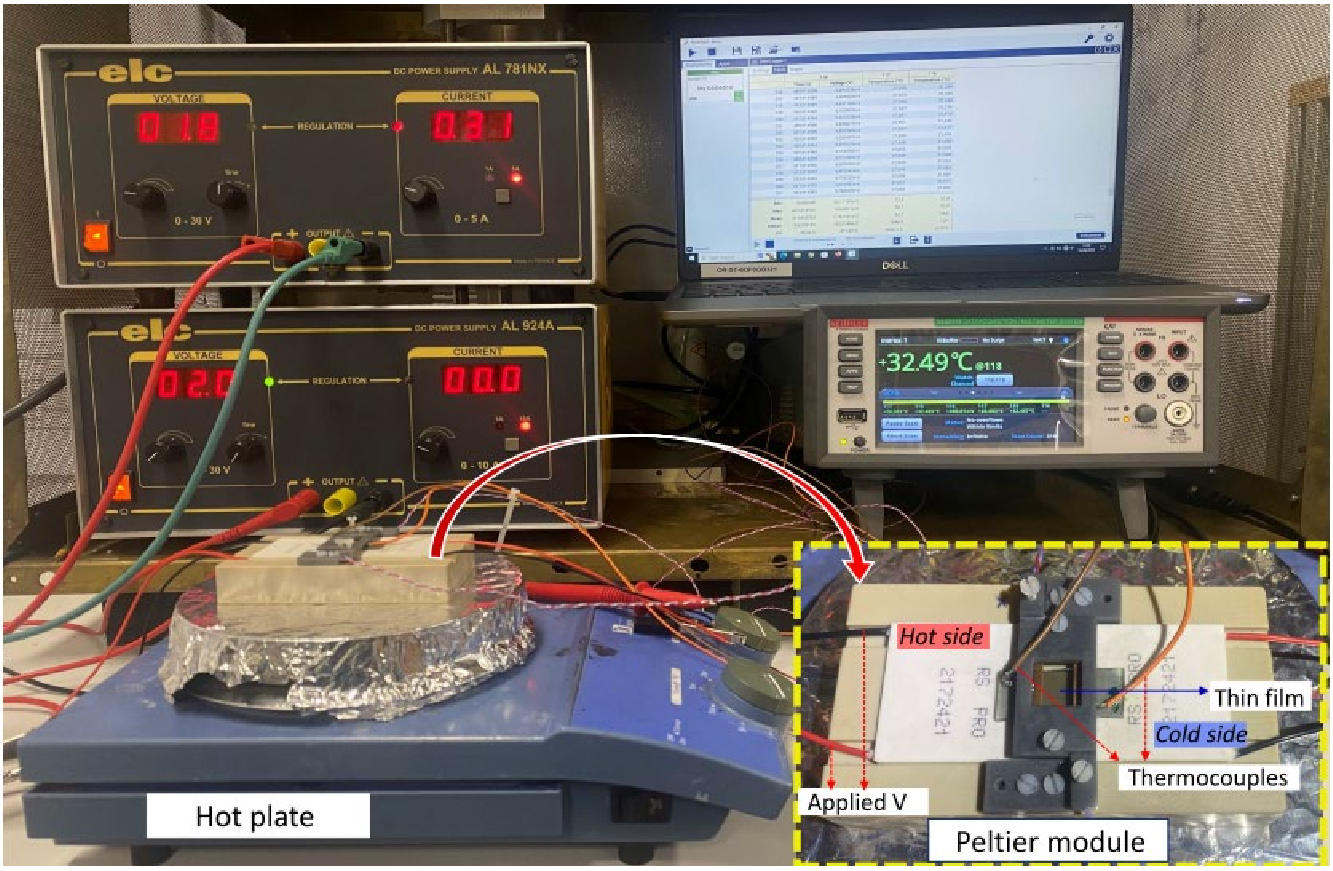

Figure S2 Peltier module based-homemade Seebeck coefficient measurement with the temperature range from 300 K to 360 K.

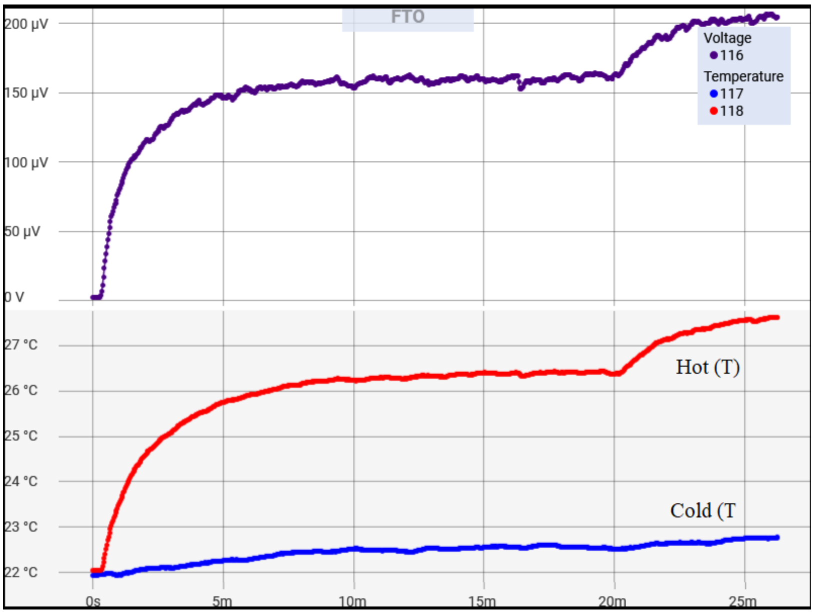

Figure S3 Example of heating sequence for establishing ΔT in a temperature range from 22 °C to 28 °C along with corresponding ΔV for FTO thin film

By varying the electrical current (I) into the Peltier module, there is a temperature variation occurs in two different sources such as hot side T_h and the cold side T_c. Data acquirement was carried out and monitored with respect to the real time. There are several parameters were recorded such as temperature (hot side and cold side), potential voltage difference (ΔV) and different acquisition times. The measurement time is relatively same for both synthesized thin films. To estimate the Seebeck coefficient (S) value, a heating sequence was performed over a temperature range from 22 °C to 28 °C (for this study; from 22 °C to 85 °C). Once T_h and T_c was stabilized, the temperature gradient ΔT (T_h - T_c) was determined followed by ΔV was estimated as shown in figure S3.
